# Ten-year change in sedentary behaviour, moderate-to-vigorous physical activity, cardiorespiratory fitness and cardiometabolic risk: independent associations and mediation analysis

**DOI:** 10.1136/bjsports-2016-096083

**Published:** 2016-08-04

**Authors:** Sara Knaeps, Jan G Bourgois, Ruben Charlier, Evelien Mertens, Johan Lefevre, Katrien Wijndaele

**Affiliations:** 1 Physical Activity, Sports and Health Research Group, Department of Kinesiology, KU Leuven, Leuven, Belgium; 2 Department of Movement and Sports Sciences, Ghent University, Ghent, Belgium; 3 Department of Human Biometrics and Biomechanics, Vrije Universiteit Brussel, Brussels, Belgium; 4 MRC Epidemiology Unit, Institute of Metabolic Science, University of Cambridge School of Clinical Medicine, Cambridge, UK

**Keywords:** Sedentary, Physical activity, Physical fitness, Health

## Abstract

**Background:**

We aimed to study the independent associations of 10-year change in sedentary behaviour (SB), moderate-to-vigorous physical activity (MVPA) and objectively measured cardiorespiratory fitness (CRF), with concurrent change in clustered cardiometabolic risk and its individual components (waist circumference, fasting glucose, high-density lipoprotein (HDL) cholesterol, triglycerides and blood pressure). We also determined whether associations were mediated by change in CRF (for SB and MVPA), waist circumference (for SB, MVPA and CRF) and dietary intake (for SB).

**Methods:**

A population-based sample of 425 adults (age (mean±SD) 55.83±9.40; 65% men) was followed prospectively for 9.62±0.52 years. Participants self-reported SB and MVPA and performed a maximal cycle ergometer test to estimate peak oxygen uptake at baseline (2002–2004) and follow-up (2012–2014). Multiple linear regression and the product of coefficients method were used to examine independent associations and mediation effects, respectively.

**Results:**

Greater increase in SB was associated with more detrimental change in clustered cardiometabolic risk, waist circumference, HDL cholesterol and triglycerides, independently of change in MVPA. Greater decrease in MVPA was associated with greater decrease in HDL cholesterol and increase in clustered cardiometabolic risk, waist circumference and fasting glucose, independent of change in SB. Greater decrease in CRF was associated with more detrimental change in clustered cardiometabolic risk and all individual components. Change in CRF mediated the associations of change in SB and MVPA with change in clustered cardiometabolic risk, waist circumference and, only for MVPA, HDL cholesterol. Change in waist circumference mediated the associations between change in CRF and change in clustered cardiometabolic risk, fasting glucose, HDL cholesterol and triglycerides.

**Conclusions:**

A combination of decreasing SB and increasing MVPA, resulting in positive change in CRF, is likely to be most beneficial towards cardiometabolic health.

## Introduction

In modern societies, a growing proportion of adults are inactive.^1^ ^2^ In addition, sedentary behaviour (SB; awake time sitting/reclining) is highly prevalent, even in those who are sufficiently active.^3^ ^4^ In recent years, accumulating evidence has suggested that excessive sitting and insufficient moderate-to-vigorous physical activity (MVPA) may independently contribute to unhealthier cardiometabolic risk profiles, which in turn may substantially increase the risk for incident type 2 diabetes, cardiovascular disease and premature death.^5^

Another factor recognised as a strong predictive marker for cardiometabolic health, and potential mediator in the associations between MVPA, SB and cardiometabolic health, is cardiorespiratory fitness (CRF).^6–8^ Developing more insight into whether, and to what extent, changes in sitting, MVPA and CRF independently shape cardiovascular health is therefore important, in order to advance lifestyle interventions and public health guidance. However, longitudinal studies examining all three potentially important lifestyle components (SB, MVPA and CRF) in this context are scarce.^9–12^ Moreover, none fully examined the potential contribution change each of these three components could make in terms of cardiometabolic health, in relation to each other.^9–11^ Change in MVPA and potentially change in SB are adjustable determinants for change in CRF.^13^ ^14^ Examining whether change in CRF mediates associations between MVPA, SB and cardiometabolic health is therefore of interest, but rarely done.^7^ Furthermore, all previous studies focused on a select population (men;^9^ ^10^ people with type 2 diabetes^11^) had a much shorter follow-up^10^ ^11^ and did not perform a maximal exercise test.^11^

Waist circumference and nutritional intake are both causally related to cardiometabolic health.^15^ ^16^ Lower MVPA and higher SB may be associated with higher waist circumference^17^ ^18^ and higher SB, especially TV viewing, is associated with increased snacking behaviour and changes in nutritional intake in general.^19–21^ Consequently, in order to better understand the mechanisms underlying any independent associations found between change in SB, MVPA and CRF, and cardiometabolic risk, change in waist circumference and nutritional intake (the latter specifically for SB) are also important candidates to examine as potential mediators. Previous longitudinal research, examining the relationship between all three exposures and cardiometabolic health, has not specifically examined the mediating role of change in waist circumference or nutritional intake.^9–11^

The purpose of the current study therefore was to: (1) examine the independent associations between change in SB, MVPA and objectively measured CRF with concurrent change in cardiometabolic risk over a long period of follow-up, and (2) to examine whether any such independent associations were mediated by change in CRF (for SB and MVPA), change in waist circumference (for SB, MVPA and CRF) or nutritional intake (for SB).

## Methods

### Participants and study design

We examined this in the longitudinal Flemish Policy Research Centre Sport study, a prospective cohort of Flemish adults aged 18–75. The sampling procedure is previously described by Duvigneaud *et al*.^22^ In 2012–2014, all volunteers who visited the examination centre in 2002–2004 (n=1569) were reinvited for follow-up, and 652 (42%) volunteers returned to participate. Written informed consent was obtained from all participants and the study was approved by the Medical Ethics Committee of the KU Leuven (s54083).

### SB and MVPA

SB and physical activity were assessed using the Flemish Physical Activity Computerized Questionnaire (FPACQ). This questionnaire generates estimates with high reliability (intraclass correlations ranging from 0.71 to 0.99) and reasonable criterion validity (correlations ranging from 0.49 to 0.57 with an objective criterion).^23^ ^24^

SB (hours/week) was estimated by asking participants to report the amount of time they spent in screen time and passive transportation. MVPA (hours/week) was estimated as a combination of sports participation and active transportation, only including activities with an intensity ≥3 metabolic equivalents (METs), according to the Ainsworth compendium.^25^

### Cardiorespiratory fitness

CRF was defined as peak oxygen uptake (VO_2peak_) relative to total body weight and determined by means of a maximal exercise test on a cycle ergometer (Lode, Groningen, the Netherlands).^26^ Oxygen consumption (VO_2_) was measured directly using a Cortex MetaLyzer 3B analyzer (Cortex Biophysic GmbH, Leipzig, Germany).^22^ ^27^ Participants first had to pass a physical examination and people at risk for heart failure and arterial hypertension did not participate in the maximal exercise test.

### Clustered cardiometabolic risk

A clustered cardiometabolic risk score (CMRS) was calculated.^28^ As described in detail before,^29^ metabolic parameters were assessed after an overnight fast. Waist circumference was measured to the nearest 0.1 cm. Systolic and diastolic blood pressures were measured by electronic monitor (Omron, the Netherlands). Triglycerides, plasma glucose and high-density lipoprotein (HDL) cholesterol were obtained from an antecubital vein blood sample and analysed by enzymatic methods (Abbott Laboratories, Abbott Park, Illinois, USA). Owing to skewness, values for the latter three parameters were first normalised (log_10_). Subsequently, standardised values for waist circumference, triglyceride, plasma glucose, blood pressure and the inverse of HDL cholesterol were computed. Each cardiometabolic variable was standardised by using the sex-specific baseline sample mean and SD, derived from all men and women with baseline data for each cardiometabolic variable. To calculate the CMRS, the sum of these standardised values was divided by the number of metabolic parameters included. In order to perform a mediation analysis considering change in abdominal adiposity as the mediator, the CMRS was also calculated without the adiposity component.^30^

### Covariates

Smoking behaviour was assessed using the WHO Monica Smoking Questionnaire. Participants were classified as current, former or never smokers. Education level was used as an indicator of socioeconomic status. Sugar intake, fat intake and alcohol consumption were assessed using a 3-day diet record, during two weekdays and one weekend day. The diet records were analysed using Becel Nutrition software (Unilever Co., Rotterdam, the Netherlands). Furthermore, the Healthy Eating Index (HEI), which captures the key recommendations of the 2010 Dietary Guidelines, was calculated.^31^

### Statistical analysis

A dropout analysis was performed by comparing baseline characteristics between the follow-up group and the group that participated at baseline only (unpaired Student's t-test and χ² test). Descriptive characteristics of the included sample were compared between baseline and follow-up (paired Student's t-test) and changes in characteristics were calculated as follow-up minus baseline.

Multiple linear regression was used to examine the association between change in the exposure variables (SB, MVPA and CRF) with change in cardiometabolic risk over 10 years. All variables were tested for normality and residuals were tested for homoscedasticity, linearity and independence. Furthermore, the variance inflation factor never exceeded two, indicating that multicollinearity was not a concern.^32^

Both exposure and outcome variables were standardised and standardised regression coefficients (95% CI) are presented, in order to enable direct comparison of the effect estimates across outcome and exposure variables (unstandardised coefficients are provided in online supplementary tables). All models were adjusted for age, sex, follow-up time, baseline value of the exposure under study, baseline value of the outcome under study; and baseline and change values in HEI, smoking and education level (model 1). Subsequently, model 1 for SB was further adjusted for change and baseline in MVPA and *vice versa* (model 2; see visual representation in online supplementary 1). All analyses were tested for an interaction effect with sex. Interaction effects between change in the exposures were also examined (ie, change in SB×change in MVPA, change in MVPA×change in CRF and change in SB×change in CRF).

10.1136/bjsports-2016-096083.supp1 supplementary data

To examine whether change in CRF mediated the associations found for SB and MVPA, a mediation analysis was performed on all significant associations between change in SB or MVPA and change in cardiometabolic risk. Finally, the mediating role of change in waist circumference (for change in SB, MVPA and CRF) and in dietary intake (only for change in SB) was also examined. All mediation analyses were performed by the product of coefficients (a×b) method by MacKinnon *et al*.^33^ The regression coefficient between the exposure and mediator (a) was adjusted for all covariates in model 2 (model 1 in case of CRF). The regression coefficient between the mediator and outcome (b) was adjusted for all covariates in model 2 (model 1 in case of CRF) and change in the relevant exposure. CIs for the mediated effect were derived with bootstrapping analysis based on 2000 repetitions.

Statistical analyses were performed using SAS, V.9.4 (SAS institute, Cary, North Carolina, USA) and Stata/SE 13.0 (Stata, College Station, Texas, USA) statistical programs.

## Results

### Descriptives

Of all 652 participants, full data were available at baseline and follow-up for 524 participants (65% men), with the exception of CRF data for which a subsample of 399 participants (64% men) provided all data. The average follow-up time was 9.62 (±0.52) years. Dropout analysis showed that more men than women remained in analyses. Furthermore, participants who returned for follow-up had higher CRF, MVPA and systolic blood pressure. They were more highly educated and reported higher sugar, saturated fat and fibre intake. No differences between both samples were found for SB or for any of the other outcomes and covariates.

Table 1 presents descriptive statistics of the included sample. Cardiometabolic risk deteriorated for almost all markers, and so did CRF. SB increased by, on average, 2 hours a week, whereas total MVPA stagnated. At follow-up, 43% of participants never smoked, 48% were former smokers and 9% were current smokers, where 71% did not change their smoking behaviour since baseline. Almost 60% of the participants had at least a professional bachelor's degree at follow-up, and education level improved in 16 participants. Table 2 presents a correlation matrix for baseline and change in all exposure variables.

**Table 1:** Descriptive statistics for age, nutrition, physical activity, cardiorespiratory fitness and cardiometabolic markers

	Baseline		10-year follow-up		Change	
Characteristics (n=524)	M	SD	M	SD	M	SD
Age (years)	46.13	9.54	55.83	9.40	9.70*	0.51
Nutrition						
Sugar intake (g/day)	82.28	8.59	74.74	40.70	−7.55*	43.21
Saturated fat intake (g/day)	37.05	15.12	34.86	14.75	−2.19**	15.63
Fibre intake (g/day)	23.58	43.46	23.87	8.17	0.30	8.34
Alcohol intake (g/day)	14.08	15.01	14.64	15.40	0.56	13.48
Total energy intake (kcal)	2405	680	2274	632	−130*	662
Healthy Eating Index	46.43	10.89	48.53	10.45	2.10*	11.42
Cardiometabolic markers						
Waist circumference (cm)	84.39	10.33	85.75	10.35	1.36*	4.93
Triacylglycerol (mmol/L)	1.18	0.64	1.15	0.62	−0.03	0.57
HDL cholesterol (mmol/L)	1.58	0.39	1.54	0.38	−0.04*	0.27
Fasting plasma glucose (mmol/L)	5.10	0.49	5.13	0.61	0.03	0.50
Diastolic blood pressure (mm Hg)	79	8.27	86	9.61	7.06*	9.40
Systolic blood pressure (mm Hg)	126	13.76	135	17.29	9.20*	15.18
CMRS	0.00	0.59	0.09	0.65	0.09*	0.47
CMRS_no−adip_	0.00	0.59	0.07	0.66	0.07*	0.52
Sedentary behaviour						
Screen time (hours/week)	13.67	7.71	15.67	8.06	2.00*	7.06
Passive transportation (hours/week)	5.06	3.94	5.00	3.83	−0.06	3.99
Total (hours/week)	18.73	7.93	20.67	8.70	1.94*	7.76
Moderate-to-vigorous physical activity						
Sport participation (hours/week)	3.96	4.29	3.00	4.01	−0.95*	4.28
Active transportation (hours/week)	0.87	0.85	1.24	1.33	0.37*	1.23
Total (hours/week)	4.83	4.59	4.24	4.43	−0.59	4.58
Cardiorespiratory fitness†						
VO_2peak_ (mL/min/kg)	34.60	8.34	33.65	8.76	−1.47*	5.87

*p<0.05; **p<0.01; ***p<0.001.

†Subgroup n=399.

CMRS, cardiometabolic risk score; HDL, high-density lipoprotein; M, mean; VO_2peak_, peak oxygen uptake.

**Table 2:** Correlation matrix for baseline and change in SB, MVPA and CRF

Baseline	SB	MVPA	CRF	Change in	SB	MVPA	CRF
SB	1.00			SB	1.00		
MVPA	−0.03	1.00		MVPA	−0.03	1.00	
CRF	−0.05	0.31*	1.00	CRF	−0.13*	0.20*	1.00

Spearman correlation coefficients (r) for baseline and change in SB, MVPA and CRF.

*p<0.05.

CRF, cardiorespiratory fitness; MVPA, moderate-to-vigorous physical activity; SB, sedentary behaviour.

When comparing the total group included in main analyses (n=524) and the group with CRF data (n=399), no differences were found for MVPA, SB and most other covariates at baseline or follow-up. However, those without CRF were, on average, older, showed lower HDL cholesterol at baseline, higher triglycerides at follow-up, and higher blood pressure and waist circumference at both time points.

### Associations with cardiometabolic risk

Standardised and unstandardised linear regression coefficients (95% CI) are presented in table 3 and online supplementary 2, respectively. There were no interaction effects by sex for any of the associations; hence, all associations are shown for men and women combined. Greater increases in SB were associated with greater increases in CMRS, CMRS_no−adip_, waist circumference, triglycerides and diastolic blood pressure, and greater decreases in HDL cholesterol after adjusting for relevant confounders (model 1). Adjustment for baseline and change in MVPA in model 2 did not attenuate these associations. Change in CRF mediated the associations with change in CMRS and change in waist circumference (CMRS: a×b (95% CI) 0.002 (0.000 to 0.004); waist circumference: 0.02 (0.00 to 0.04)), but not the associations with change in HDL cholesterol, triglycerides or diastolic blood pressure. Change in waist circumference or nutritional intake did not mediate the associations with CMRS_no−adip_, HDL cholesterol, triglycerides or diastolic blood pressure.

**Table 3:** Standardised regression coefficients of sedentary behavior, MVPA and CRF for clustered cardiometabolic health and cardiometabolic markers

		Change in SB		Change in MVPA		Change in CRF	
Change in	Model		95% CI		95% CI		95% CI
CMRS	1	0.20*	0.11 to 0.28	−0.17*	−0.27 to −0.07	−0.40*	−0.49 to −0.30
	2	0.20*	0.11 to 0.29	−0.17*	−0.27 to −0.08		
CMRS_no−adip_	1	0.19*	0.10 to 0.28	−0.17*	−0.27 to −0.07	−0.34*	−0.43 to −0.24
	2	0.19*	0.10 to 0.27	−0.17*	−0.26 to −0.07		
Waist circumference	1	0.12**	0.03 to 0.21	−0.11***	−0.21 to −0.01	−0.40*	−0.50 to −0.31
	2	0.12***	0.03 to 0.21	−0.11***	−0.21 to −0.01		
Fasting glucose	1	0.01	−0.08 to 0.10	−0.10***	−0.20 to 0.00	−0.13**	−0.23 to −0.03
	2	0.01	−0.09 to 0.10	−0.10***	−0.20 to 0.00		
HDL cholesterol	1	−0.16*	−0.25 to −0.08	0.13**	0.04 to 0.23	0.23*	0.14 to 0.33
	2	−0.16*	−0.25 to −0.07	0.14**	0.04 to 0.23		
Triglycerides	1	0.15*	0.07 to 0.24	−0.09	−0.18 to 0.00	−0.21*	−0.31 to −0.11
	2	0.15*	0.07 to 0.24	−0.08	−0.17 to 0.01		
Diastolic blood pressure	1	0.12**	0.03 to 0.21	−0.08	−0.18 to 0.01	−0.22*	−0.31 to −0.13
	2	0.12**	0.04 to 0.20	−0.09	−0.18 to 0.01		
Systolic blood pressure	1	0.08	−0.01 to 0.17	0.01	−0.09 to 0.10	−0.14**	−0.22 to −0.05
	2	0.08	−0.01 to 0.17	0.00	−0.09 to 0.10		

Data are standardised regression coefficients.

Model 1: adjusted for age, follow-up time, sex, original study population; baseline and changes in healthy eating, smoking, education level; baseline of the relevant exposure and outcome (SB and MVPA n=524; CRF n=399).

Model 2: adjusted for all covariates in model 1 and adjusted for changes and baseline MVPA for SB and vice versa.

*p<0.05; **p<0.01; ***p<0.001.

CMRS, cardiometabolic risk score; CRF, cardiorespiratory fitness; HDL, high-density lipoprotein; MVPA, moderate-to-vigorous physical activity; SB, sedentary behaviour.

10.1136/bjsports-2016-096083.supp2 supplementary data

Greater decreases in MVPA were associated with greater increases in CMRS, CMRS_no−adip_, waist circumference, fasting glucose and greater decreases in HDL cholesterol (model 1). Again, adjustment for baseline and change in SB did not attenuate these associations (model 2). Similarly to results for SB, change in CRF mediated the associations with change in CMRS and waist circumference (CMRS: a×b (95% CI) −0.01 (−0.01 to −0.00); waist circumference: −0.09 (−0.14 to −0.04)). We also found evidence for CRF mediation for HDL cholesterol (0.08 (0.01 to 0.14)), but not for fasting glucose. Change in waist circumference did not mediate the association with CMRS_no−adip_, HDL cholesterol or fasting glucose.

Greater decreases in CRF were associated with greater increases in all cardiometabolic markers, except for HDL cholesterol, showing a positive association (model 1). Change in waist circumference did not mediate the associations with blood pressure. However, change in waist circumference mediated the associations with CMRS_no−adip_, fasting glucose, HDL cholesterol and triglycerides (CMRS_no−adip_: −0.01 (−0.01 to −0.01); fasting glucose: −0.11 (−0.18 to −0.04); HDL cholesterol: 0.15 (0.08 to 0.22); and triglycerides: −0.40 (−0.81 to −0.01)).

A sensitivity analysis was run to examine whether associations for change in SB and MVPA in models 1 and 2 were modified in the smaller subset of participants with CRF data (see online supplementary 3). In general, associations were equivalent in terms of strength and direction compared with those found in the larger sample. Associations between change in MVPA and change in blood pressure were more pronounced in the smaller sample.

10.1136/bjsports-2016-096083.supp3 supplementary data

**Figure 1:**
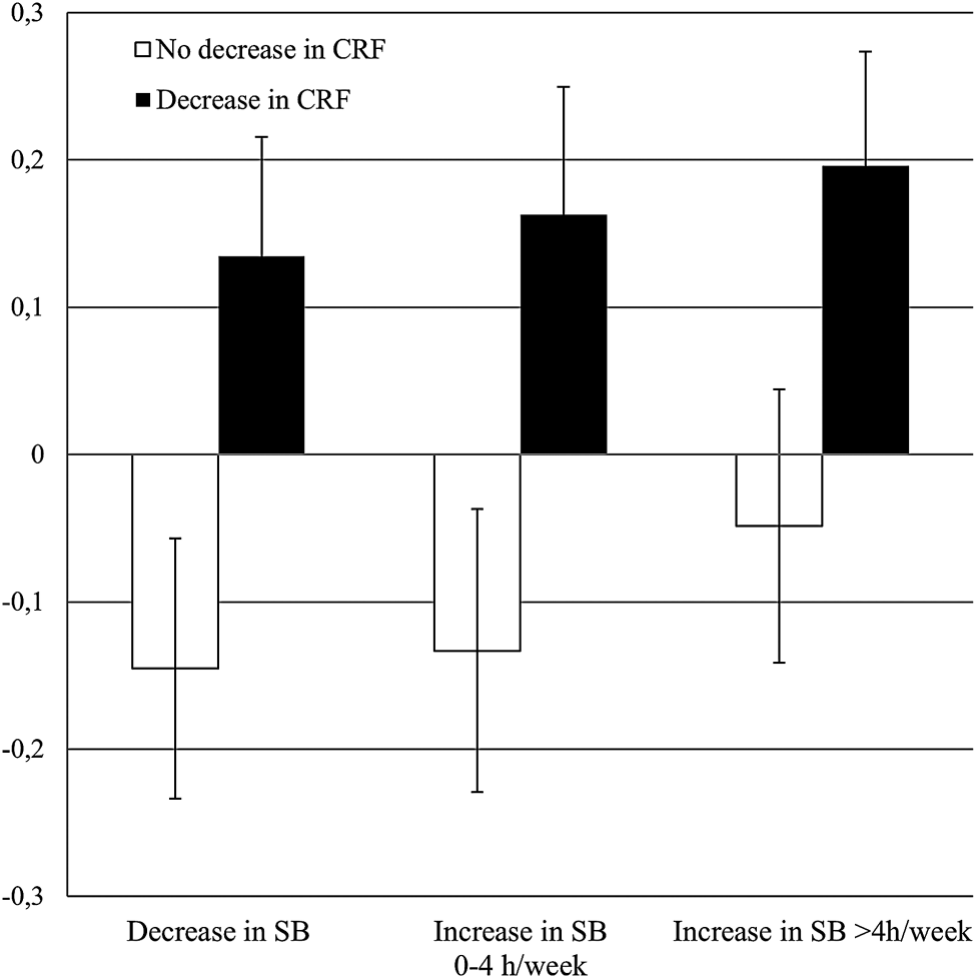
Displays the estimated marginal means (SE) for change in cardiometabolic risk score in six groups of participants, defined by their change in cardiorespiratory fitness (CRF) (decrease (62%, white bars) and no decrease (38%, black bars) over time) and change in sedentary behaviour (SB) (decrease (37%), increase between 0 and 4 hours/week (24%) and increase by >4 hours/week (39%)).

Of all possible interaction effects between change in the exposures, only one interaction, more specifically between change in SB and CRF for the association with HDL cholesterol, was borderline significant (β=−0.03; −0.05; −0.00), indicating that the associations found in table 3 did not differ by different levels of change in the other exposures. Figure 1 displays the estimated marginal means (SE) for change in CMRS in six groups of participants, defined by their change in CRF (decrease (62%, white bars) and no decrease (38%, black bars) over time) and change in SB (decrease (37%), increase between 0 and 4 hours/week (24%) and increase by >4 hours/week (39%)). The direction and strength of association between change in SB and CMRS was similar among those who increased and decreased their CRF. More specifically, in those with increasing CRF, increasing SB was associated with less decrease in clustered cardiometabolic risk, and in those with decreasing CRF, increasing SB was associated with greater increase in clustered cardiometabolic risk.

## Discussion

Our results suggest that favourable changes in self-reported SB, MVPA and objectively measured CRF are associated with favourable changes in *clustered* cardiometabolic risk, independently from confounders and also from baseline and change in MVPA and SB, as applicable. Furthermore, increases in CRF were associated with favourable changes in all *individual* cardiometabolic risk markers; a decrease in SB, independently from change in MVPA, with a decrease in waist circumference, triglycerides, diastolic blood pressure and an increase in HDL cholesterol; and an increase in MVPA, independently from change in SB, with a decrease in waist circumference, fasting glucose and an increase in HDL cholesterol. In context, owing to the dose-dependent association between plasma triglycerides and both cardiovascular and all-cause mortality, for example, decreasing sitting time over 10 years by 2 hours/day would be associated with a 1% lower relative risk for cardiovascular and all-cause mortality, in an already relatively healthy population such as the present one.^34^

Change in CRF mediated the associations between change in SB and change in CMRS and waist circumference, and the associations between change in MVPA and change in CMRS, waist circumference and HDL cholesterol. Change in waist circumference did not mediate these associations for SB or MVPA, and change in nutritional intake did not mediate the associations found for change in SB. However, change in waist circumference did mediate the association between CRF and CMRS_no−adip_, fasting glucose, HDL cholesterol and triglycerides. This means that change in waist circumference might be a biologically plausible mechanism explaining the detrimental effects of low CRF on certain cardiometabolic health parameters.

### Strengths and limitations

In this study, we were able to examine the relative importance of long-term changes in each of the three exposures for cardiometabolic health and their interrelationships. Previous longitudinal research examined individual associations between changes in SB, MVPA and CRF and clustered cardiometabolic risk,^11^ ^21^ ^35–38^ but rarely included a mediation analysis for CRF.^7^ Additional strengths of this study include the 10-year follow-up, which is longer than all previous studies.^11^ ^30^ ^36^ ^37^ ^39^ Furthermore, to reduce measurement error, CRF was measured with a maximal cycle ergometer test and objectively measured VO_2_, the gold standard for CRF testing.^26^ Measuring CRF objectively in large studies is costly, not without danger and time-consuming. Therefore, CRF is generally approximated by extrapolation of a submaximal test.^11^ ^35^ ^38^ Finally, since all analyses investigated changes within participants, interference of genetic influences is less likely.

The following limitations should, however, be considered. First, SB and MVPA were self-reported and did not cover all domains of daily living. Estimates included screen time, passive and active transportation and sports participation, and only including certain types of SB and MVPA might have introduced considerable bias. Self-report, in comparison with objective measurement, is also associated with more measurement error, increasing the risk for regression attenuation bias and potentially lowering the strength of the associations found for SB and MVPA. However, the FPACQ has been validated extensively^23^ ^24^ and shows, in comparison to other self-report measures, high test-retest reliability and criterion validity.^40^ Furthermore, change in SB and MVPA is likely to be captured with smaller measurement error compared with an estimate of these behaviours at one single time-point, as participants are likely to misreport to a similar extent at both time-points.^37^ Second, people at risk for heart failure and arterial hypertension did not participate in the maximal exercise test and therefore CRF data were only available for a healthier subgroup. However, a sensitivity analysis which examined whether associations for change in SB and MVPA were modified in the smaller subset of participants with CRF data compared with the total included participant group showed no substantial differences in associations. Furthermore, non-inclusion of these participants may have reduced the risk for reverse causality for the associations found. Nevertheless, associations found in this study are relative to the fairly small cohort of Caucasian adults included in analysis and need to be examined in other populations, heterogeneous in terms of ethnicity and health status. Third, although CMRS has several advantages for evaluating cardiometabolic risk over the dichotomous classification of the metabolic syndrome,^29^ the score is sample specific and therefore highly dependent on the distribution of risk factors of the included participants. Finally, analyses could not be controlled for medication use. Although this was a healthy adult population, some participants would have taken relevant medication, which might cause residual confounding. However, exclusion for arterial hypertension in the subsample analyses will have minimised the impact of this for antihypertensive medication.

### Comparison to previous research

To the best of our knowledge, no previous longitudinal studies examined associations between changes in SB, MVPA and CRF and cardiometabolic risk and the mediating role of CRF. However, our findings extend previous cross-sectional and longitudinal findings of associations between SB and clustered cardiometabolic risk,^9^ ^30^ ^41^ waist circumference,^30^ ^41^ HDL cholesterol^41^ and triglycerides,^30^ ^40^ independent of MVPA. Other cross-sectional^38^ ^42^ and longitudinal^10^ ^11^ studies could not confirm an association between SB and clustered cardiometabolic risk, when adjusting for MVPA.

Inconsistent findings for SB and cardiometabolic risk might be due to a number of reasons. First, in other populations with more homogeneous outcome profiles, associations with clustered cardiometabolic risk might be difficult to detect.^38^ ^42^ Second, in patient populations with higher medication use, the positive effects of lowering SB might be drowned out by the health improvements caused by the use of this medication.^11^ Third, the type and measurement method of SB under study might also cause inconsistencies. Self-reported SB typically includes mainly screen time, while studies using objective measurement include all SBs.^30^ ^43^ The mean change in SB in our study was mostly driven by a change in screen time, which may be associated with a different confounding structure and different pathways explaining associations (such as differences in dietary intake), compared with studies that implemented objective measurement of total sedentary time.^11^ ^21^ ^38^

Change in MVPA was associated with change in clustered cardiometabolic risk, waist circumference, fasting glucose and HDL cholesterol, independent from change in SB. These associations were found to be mediated through change in CRF, except for the association with fasting glucose. When comparing to a cross-sectional study, the association between MVPA and HDL cholesterol and fasting glucose, independent of SB, was of similar strength.^38^ However, for the independent association with waist circumference, this study observed a considerably smaller association.^38^ Differences in study design, statistical analysis and measurement method may have caused these differences in results.

Finally, changes in CRF were associated with changes in clustered cardiometabolic risk and all individual markers. Furthermore, these associations were almost always more than twice as strong as those with changes in SB or MVPA. The difference in strength of associations is in line with previous research, where CRF is often recognised as one of the most important determinants of longevity and health.^7^ ^9^ ^44^ However, part of this difference in strength of association might be due to the difference in measurement error between objectively measured CRF and self-reported SB and MVPA. Furthermore, CRF is a physiological measure reflecting a combination of genetic potential, behavioural and functional health of various organ systems.^45^ As a result, CRF improves mostly when MVPA increases and when SB decreases.^46^ Therefore, CRF was also examined as a potential mediator in all significant associations between change in SB or MVPA and change in cardiometabolic health.

## Conclusions

In conclusion, favourable changes in self-reported SB and MVPA are independently associated with positive changes in clustered cardiometabolic health, and favourable changes in CRF are associated with positive changes in clustered cardiometabolic health. The associations between change in SB and MVPA and change in clustered cardiometabolic risk were mediated through changes in CRF and the association between CRF and clustered cardiometabolic risk was mediated through changes in waist circumference. On the basis of these results, a combination of decreasing SB and increasing MVPA, most likely resulting in a positive change in CRF, is most beneficial towards cardiometabolic health. Further longitudinal studies investigating changes in all three exposures measured objectively are necessary to gain better insights into their associations and inter-relationships with cardiometabolic health as well as intervention studies that try to integrate both SB and MVPA and evaluate the mediating effect of CRF.

**What are the findings?**

- The greater increase in sedentary behaviour (SB) and decrease in moderate-to-vigorous physical activity (MVPA) was associated with greater increase in clustered cardiometabolic risk, independently from change in MVPA and SB, respectively.
- The greater decrease in cardiorespiratory fitness (CRF) was associated with greater increase in clustered cardiometabolic risk.
- The associations of change in SB and MVPA with change in clustered cardiometabolic risk were mediated through change in CRF.
- The association between change in CRF and change in clustered cardiometabolic risk was mediated through change in waist circumference.

**How might it impact on clinical practice in the future?**

- Clinical practice should recommend lifestyle changes resulting in a combination of decreasing SB and increasing MVPA, most likely resulting in a positive change in CRF, for the most beneficial cardiometabolic health.
