# Supplementary material for: Ten-year change in sedentary behaviour, moderate-to-vigorous physical activity, cardiorespiratory fitness and cardiometabolic risk: independent associations and mediation analysis
Source: Br J Sports Med. 2016 Aug 4;52(16):1063–8. doi: 10.1136/bjsports-2016-096083 (PMC6089204; doi:10.1136/bjsports-2016-096083)
Supplement: supplementary data [file bjsports-2016-096083supp001.pdf]

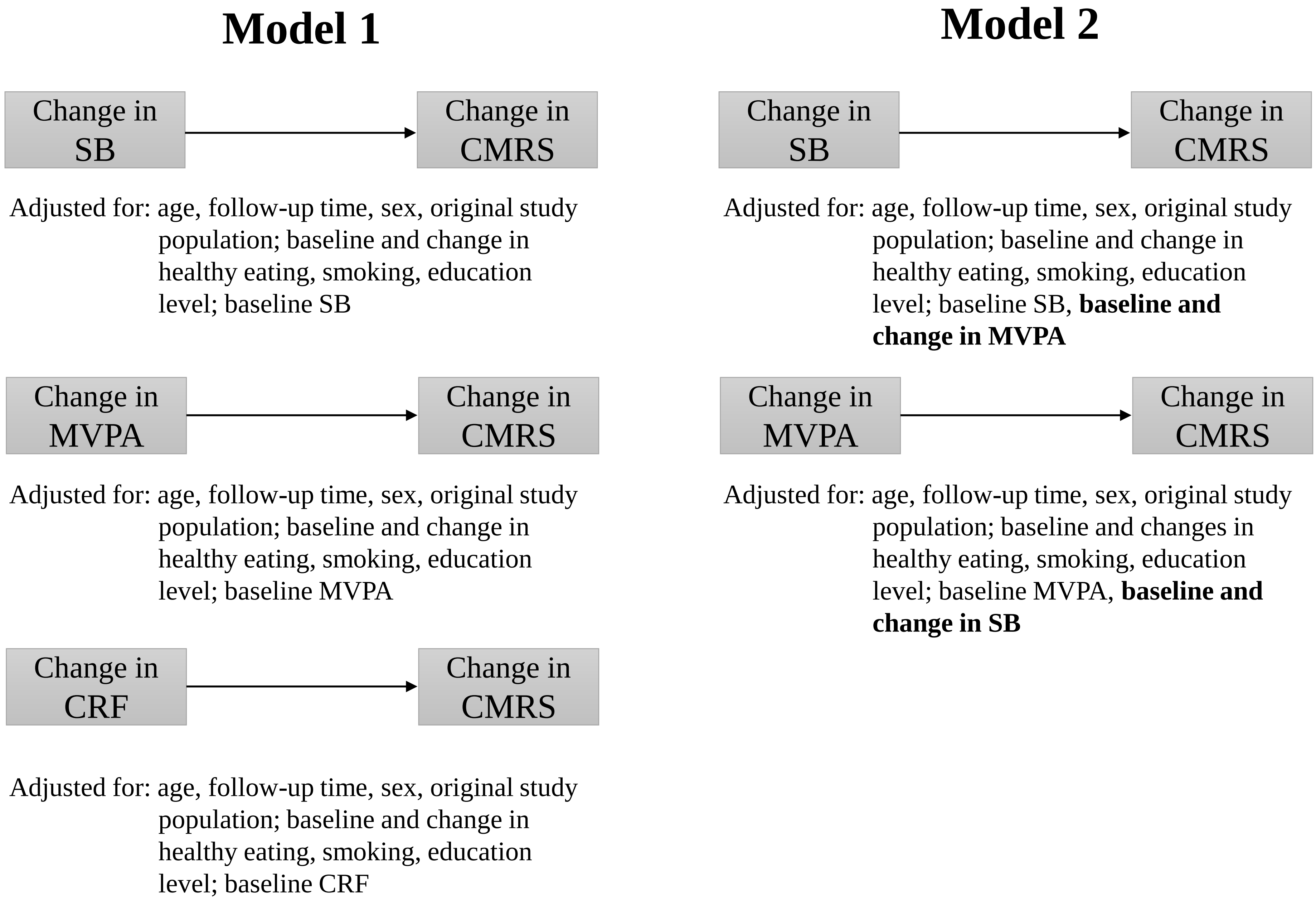

Note. SB = Sedentary Behavior; MVPA = moderate-to-vigorous physical activity; CRF=Cardiorespiratory fitness, CMRS = Cardio-metabolic risk score
