# Supplementary material for: Ten-year change in sedentary behaviour, moderate-to-vigorous physical activity, cardiorespiratory fitness and cardiometabolic risk: independent associations and mediation analysis
Source: Br J Sports Med. 2016 Aug 4;52(16):1063–8. doi: 10.1136/bjsports-2016-096083 (PMC6089204; doi:10.1136/bjsports-2016-096083)
Supplement: supplementary data [file bjsports-2016-096083supp002.pdf]

Supplement 1. Regression coefficients of sedentary behavior, moderate-to-vigorous physical activity and cardiorespiratory fitness for clustered cardio-metabolic health and cardio-metabolic markers

Change in	Model	Change in SB		Change in MVPA		Change in CRF	
			95% CI		95% CI		95% CI
CMRS	1	0.01 [c]	0.01 , 0.02	-0.02 [c]	-0.03 , -0.01	-0.03 [c]	-0.04 , -0.02
	2	0.01 [c]	0.01 , 0.02	-0.02 [c]	-0.03 , -0.01		
CMRS$_{no\text{-}adip}$	1	0.01 [c]	0.01 , 0.02	-0.02 [c]	-0.03 , -0.01	-0.03 [c]	-0.04 , -0.02
	2	0.01 [c]	0.01 , 0.02	-0.02 [c]	-0.03 , -0.01		
Waist Circumference (cm)	1	0.07 [b]	0.02 , 0.13	-0.12 [a]	-0.22 , -0.01	-0.34 [c]	-0.42 , -0.26
	2	0.08 [a]	0.02 , 0.13	-0.12 [a]	-0.23 , -0.01		
Fasting Glucose (mmol/L)	1	0.00	-0.01 , 0.01	-0.01 [a]	-0.02 , 0.00	-0.01 [b]	-0.02 , 0.00
	2	0.00	-0.01 , 0.01	-0.01 [a]	-0.02 , 0.00		
HDL-cholesterol (mmol/L)	1	-0.01 [c]	-0.01 , 0.00	0.01 [b]	0.00 , 0.01	0.01 [c]	0.01 , 0.02
	2	-0.01 [c]	-0.01 , 0.00	0.01 [b]	0.00 , 0.01		
Triglycerides (mmol/L)	1	0.01 [c]	0.01 , 0.02	-0.01	-0.02 , 0.00	-0.02 [c]	-0.03 , -0.01
	2	0.01 [c]	0.01 , 0.02	-0.01	-0.02 , 0.00		
Diastolic Blood Pressure (mmHg)	1	0.14 [b]	0.04 , 0.24	-0.17	-0.37 , 0.02	-0.35 [c]	-0.50 , -0.21
	2	0.14 [b]	0.03 , 0.24	-0.18	-0.37 , 0.01		
Systolic Blood Pressure (mmHg)	1	0.16	-0.01 , 0.32	0.02	-0.29 , 0.34	-0.35 [b]	-0.57 , -0.13
	2	0.15	-0.02 , 0.32	0.02	-0.30 , 0.33		

Legend Supplement 1

SB = Sedentary Behavior; MVPA = moderate-to-vigorous physical activity; CRF=Cardiorespiratory fitness

Model 1: adjusted for age, follow-up time, sex, original study population; baseline and changes in healthy eating, smoking, education level; baseline of the relevant exposure and outcome (SB & MVPA n=524; CRF n=399)

Model 2: adjusted for all covariates in model 1 adjusted for changes and baseline MVPA for SB and vice versa (n=524)

[a] $p<0.05$; [b] $p<0.01$ [c] $p<0.001$
