# Supplementary material for: Ten-year change in sedentary behaviour, moderate-to-vigorous physical activity, cardiorespiratory fitness and cardiometabolic risk: independent associations and mediation analysis
Source: Br J Sports Med. 2016 Aug 4;52(16):1063–8. doi: 10.1136/bjsports-2016-096083 (PMC6089204; doi:10.1136/bjsports-2016-096083)
Supplement: supplementary data [file bjsports-2016-096083supp003.pdf]

Supplement 2. Standardized regression coefficients of sedentary behavior, moderate-to-vigorous physical activity and cardiorespiratory fitness for clustered cardio-metabolic health and cardio-metabolic markers in a subgroup with complete data

		Change in SB		Change in MVPA		Change in CRF	
Change in	Model		95% CI		95% CI		95% CI
CMRS	1	0.19 [c]	0.08 , 0.29	-0.21 [c]	-0.32 , -0.10	-0.40 [c]	-0.49 , -0.30
	2	0.18 [c]	0.08 , 0.29	-0.21 [c]	-0.32 , -0.10		
CMRS$_{no\text{-}adip}$	1	0.18 [c]	0.07 , 0.28	-0.20 [c]	-0.31 , -0.09	-0.34 [c]	-0.43 , -0.24
	2	0.17 [c]	0.07 , 0.28	-0.20 [c]	-0.31 , -0.09		
Waist Circumference	1	0.11 [a]	0.01 , 0.22	-0.15 [a]	-0.26 , -0.03	-0.40 [c]	-0.50 , -0.31
	2	0.11 [a]	0.01 , 0.22	-0.15 [a]	-0.26 , -0.03		
Fasting Glucose	1	0.04	-0.06 , 0.15	-0.05	-0.16 , 0.06	-0.13 [b]	-0.23 , -0.03
	2	0.04	-0.06 , 0.15	-0.05	-0.16 , 0.07		
HDL-cholesterol	1	-0.12 [a]	-0.22 , -0.02	0.22 [c]	0.11 , 0.32	0.23 [c]	0.14 , 0.33
	2	-0.12 [a]	-0.22 , -0.02	0.21 [c]	0.10 , 0.32		
Triglycerides	1	0.14 [b]	0.04 , 0.24	-0.09	-0.20 , 0.01	-0.21 [c]	-0.31 , -0.11
	2	0.14 [b]	0.04 , 0.24	-0.09	-0.20 , 0.01		
Diastolic Blood Pressure	1	0.10 [a]	0.00 , 0.20	-0.14 [a]	-0.25 , -0.03	-0.22 [c]	-0.31 , -0.13
	2	0.11 [a]	0.01 , 0.21	-0.14 [a]	-0.24 , -0.03		
Systolic Blood Pressure	1	0.08	-0.02 , 0.18	-0.06	-0.17 , 0.04	-0.14 [b]	-0.22 , -0.05
	2	0.09	-0.01 , 0.19	-0.06	-0.17 , 0.04		

Legend Supplement 2

Data are standardized regression coefficients

SB = Sedentary Behavior; MVPA = moderate-to-vigorous physical activity; CRF=Cardiorespiratory fitness

Model 1: adjusted for age, follow-up time, sex, original study population; baseline and changes in healthy eating, smoking, education level; baseline of the relevant exposure and outcome (SB, MVPA & CRF n=399)

Model 2: adjusted for all covariates in model 1 adjusted for changes and baseline MVPA for SB and vice versa;

[a] $p<0.05$; [b] $p<0.01$; [c] $p<0.001$
